# Impact of Traditional Healers on the HIV Care Cascade in Senegal, West Africa: A Longitudinal Study

**DOI:** 10.4269/ajtmh.21-0280

**Published:** 2021-08-23

**Authors:** Noelle A. Benzekri, Jacques F. Sambou, Sanou Ndong, Mouhamadou Baïla Diallo, Ibrahima Tito Tamba, Dominique Faye, Ibrahima Sall, Jean Philippe Diatta, Khadim Faye, Fatima Sall, Ousseynou Cisse, Cheikh T. Ndour, Papa Salif Sow, Jean Jacques Malomar, Stephen E. Hawes, Moussa Seydi, Geoffrey S. Gottlieb

**Affiliations:** ^1^ Department of Medicine, University of Washington, Seattle, Washington;; ^2^ Centre de Santé de Ziguinchor, Ziguinchor, Senegal;; ^3^ Services des Maladies Infectieuses et Tropicales, Centre Hospitalier National Universitaire de Fann, Dakar, Senegal;; ^4^ Centre de Santé de Bignona, Bignona, Senegal;; ^5^ Division de Lutte contre le Sida et les Infections Sexuellement Transmissibles, Ministère de la Santé et de l’Action Sociale, Dakar, Senegal;; ^6^ Department of Epidemiology, University of Washington, Seattle, Washington;; ^7^ Department of Global Health, University of Washington, Seattle, Washington

## Abstract

Consultation with traditional healers (THs) is common among people living with HIV in sub-Saharan Africa. We conducted a prospective longitudinal study to determine the association between consultation with THs and HIV outcomes following 12 months of antiretroviral therapy (ART). HIV-infected individuals presenting for care and initiation of ART in Dakar and Ziguinchor, Senegal were eligible for enrollment. Data were collected using interviews, clinical evaluations, laboratory analyses, and chart reviews at enrollment, 6 months after ART initiation, and 12 months after ART initiation. Among the 186 participants, 35.5% consulted a TH. The most common reason for consulting a TH was “mystical” concerns (18%). Those who consulted a TH before ART initiation were more likely to present with a CD4 count < 200 cells/mm^3^ (44% versus 28%; *P* = 0.04) and WHO stage 3 or 4 disease (64% versus 46%; *P* = 0.03), and they were less likely to disclose their HIV status (44% versus 65%; *P* = 0.04). Those who consulted a TH more than 6 months after ART initiation were more likely to report poor adherence to ART (57% versus 4%; *P* < 0.01). The strongest predictor of virologic failure was consulting a TH more than 6 months after ART initiation (odd ratio [OR], 7.43; 95% CI, 1.22–45.24). The strongest predictors of mortality were consulting a TH before ART initiation (OR, 3.53; 95% CI, 1.25–9.94) and baseline CD4 count < 200 cells/mm^3^ (OR, 3.15; 95% CI, 1.12–8.89). Our findings reveal multiple opportunities to strengthen the HIV care cascade through partnerships between THs and biomedical providers. Future studies to evaluate the impact of these strategies on HIV outcomes are warranted.

## INTRODUCTION

Understanding and addressing the persistent social and cultural barriers to HIV testing and treatment is critical to strengthening the HIV care cascade and ending the HIV epidemic. Despite significant biomedical advancements in the prevention, diagnosis, and treatment of HIV, there were nearly 1 million individuals infected with HIV in sub-Saharan Africa (SSA) in 2019 and 440,000 deaths due to AIDS.^1^ In Western and Central Africa, only 68% of people living with HIV (PLHIV) know their HIV status, 58% are receiving antiretroviral therapy (ART), and only 45% are virally suppressed.^1^

According to the WHO, approximately 80% of individuals in SSA use traditional medicine, and for many, including PLHIV, the traditional healer (TH) represents their primary source of health care.^2^^–^^8^ As such, the WHO has advocated for the integration of THs into national HIV programs and for collaboration between traditional healers and biomedical care providers.^2^^,^^5^^,^^9^

THs in SSA reflect the great diversity of cultures and belief systems found on the African continent^2^ and include “herbalists, spiritualists, diviners or any other practitioner trained or gifted in these forms of healing and recognized as such by the community”^5^ Understanding how consultation with THs impacts HIV outcomes could contribute to the development of high-impact strategies to partner with THs and improve the HIV care cascade. We previously reported an association between seeking care from THs before the initiation of ART and mortality among PLHIV in Senegal, West Africa.^10^ Here we report the results of a prospective longitudinal study to determine the association between consultation with THs and HIV outcomes 12 months after ART initiation.

## METHODS

HIV-positive individuals presenting for care and initiation of ART at the Clinique des Maladies Infectieuses, Center Hospitalier National Universitaire de Fann in Dakar, Senegal, and the Center de Santé de Ziguinchor, Senegal, were eligible for enrollment. Informed consent was conducted by the social worker. For participants younger than 18 years of age, consent was obtained from their legal guardian. Study procedures were approved by the University of Washington Institutional Review Board and the Senegal Comité National d’Ethique pour la Recherche en Santé.

Data were collected using semi-structured interviews, clinical evaluations, laboratory analyses, and chart review at enrollment, 6 months after ART initiation, and 12 months after ART initiation. At each study encounter, participants were asked if they had consulted with a TH. Participants were categorized according to history of consultation with a TH prior to initiation of ART, after initiation of ART, or after 6 months of ART. They were also asked the reason for consulting a TH and the date of the most recent consultation with a TH.

ART adherence was determined by participant report. Participants were asked how many times in the past 7 days they failed to take one or more doses of ART, how many times in the past 7 days they failed to take all of their doses of ART, how many times in the past 4 weeks they failed to take one or more doses of ART, and how many times in the past 4 weeks they failed to take all of their doses of ART. They were also asked if they were “always adherent” to their ART, “not always adherent” to their ART, or “not adherent” to their ART. Those who took less than 90% of their ART or responded “not always adherent” or “not adherent” were classified as poorly adherent.

HIV-associated outcomes were determined at the end of the 12 month follow-up period. HIV-1 and HIV-2 plasma RNA viral loads were measured using real-time HIV-1 and HIV-2 Abbott m2000 platform assays.^11^ Virologic failure was defined as > 1,000 copies/mL for HIV-1 and > 250 copies/mL for HIV-2. Medical records and family reports were used to ascertain mortality. Patients who had no contact with the clinic for more than 6 months and could not be traced by telephone or home visit were considered lost to follow-up.

Data were analyzed using SPSS Statistics 26 (IBM SPSS, Armonk, NY). Descriptive analysis was performed for all variables. The χ^2^ and Fisher’s exact tests were used to identify differences in outcomes between participants who had consulted a TH and those who had not consulted a TH. Logistic regression analyses were used to identify predictors of virologic failure and mortality. Factors identified as significant by simple regressions and predictors previously identified in the literature were included in multivariable regression analyses. Participants with missing outcome data were excluded from the final analysis. *P* < 0.05 was considered significant.

## RESULTS

We enrolled 186 HIV-positive participants in this study, of which 66 (35%) consulted a traditional healer prior to ART or during follow-up; 58 participants (33%) consulted a traditional healer prior to initiation of ART, 15 (12%) consulted a traditional healer after initiation of ART, and 7 (7%) consulted a traditional healer after 6 months of ART (Table 1). Of those who consulted a TH, 70% were enrolled at the Ziguinchor site.

**Table 1:** Characteristics of 186 HIV-infected individuals in Senegal who consulted a traditional healer (TH) before antiretroviral therapy (ART) or after ART initiation compared to those who did not consult a TH

	All participants	Consulted TH^*^	Did not consult TH	*P* value
	*n* (%)	*n* (%)	*n* (%)	
Total number of participants	186 (100)	66 (35.5)	120 (64.5)	
Enrollment site				0.02
Ziguinchor	108 (58.1)	46 (69.7)	62 (51.7)	
Dakar	78 (41.9)	20 (30.3)	58 (48.3)	
Female	130 (69.9)	41 (62.1)	89 (74.2)	0.09
Age, median years (IQR)	37 (31–46)	42 (33–48)	35 (29–45)	0.01
Age categories, years of age				0.07
< 30	40 (22.1)	8 (12.5)	32 (27.4)	
30–45	96 (53.0)	37 (57.8)	59 (50.4)	
> 45	45 (24.9)	19 (29.7)	26 (22.2)	
Born in Senegal	148 (80.0)	57 (86.4)	91 (76.5)	0.11
Highest educational level obtained				0.53
No formal education	52 (31.9)	23 (37.1)	29 (28.7)	
Any primary school	64 (39.3)	23 (37.1)	41 (40.6)	
Any secondary school	47 (28.8)	16 (25.8)	31 (30.7)	
Employed	25 (14.5)	13 (21.0)	12 (10.8)	0.07
Marital status				0.08
Single	23 (12.7)	9 (13.8)	14 (12.1)	
Married	98 (54.1)	28 (43.1)	70 (60.3)	
Divorced	32 (17.7)	17 (26.2)	15 (12.9)	
Widowed	28 (15.5)	11 (16.9)	17 (14.7)	
Number of children				0.87
0–2	96 (53.6)	34 (54.0)	62 (53.4)	
3–5	60 (33.5)	22 (34.9)	38 (32.8)	
≥ 6	23 (12.8)	7 (11.1)	16 (13.8)	
Household size				0.89
1–4	40 (22.6)	13 (20.6)	27 (23.7)	
5–8	64 (36.2)	23 (36.5)	41 (36.0)	
≥ 9	73 (41.2)	27 (42.9)	46 (40.4)	
Transportation time to clinic ≥ 90 minutes	63 (35.0)	17 (27.0)	46 (39.3)	0.10
Transportation cost, round trip, median USD (IQR)	$1.74 ($0.70–$1.74)	$1.74 ($0.52–$1.78)	$1.74 ($0.70–$1.74)	0.67
Transportation cost ≥ $1.75	42 (24.0)	15 (24.2)	27 (23.9)	0.97

IQR = interquartile range; USD = United States dollars.

* Includes 58 (32.8%) prior to M0, 15 (11.5%) after M0, and 7 (6.5%) after M6; the sum is greater than 66 because the categories are not mutually exclusive.

The median age of the participants was 37 years (interquartile range [IQR], 41–46 years), and 70% were female. A greater percentage of those who consulted a TH were older than 45 years (30%) compared with those who never consulted a TH (22%), and fewer were younger than 30 years (13% versus 27%) (*P* = 0.07). The majority of participants (80%) were born in Senegal. Approximately one-third (32%) had not received any formal education, 39% had attended primary school, and 29% had attended secondary school. Only 15% of participants were formally employed. Thirteen percent were single, 54% were married, 18% were divorced, and 16% were widowed. More than half (54%) had 0–2 children and 13% had ≥6 children. Approximately 41% lived in a household with nine or more household members. More than one-third (35%) spent 90 minutes or more traveling to and from the clinic, and the median cost of transportation was $1.74. There were no significant differences in sex, birthplace, educational level, employment, marital status, number of children, household size, transportation time, or transportation cost among those who consulted a TH compared to those who never consulted a TH.

**Figure 1.**
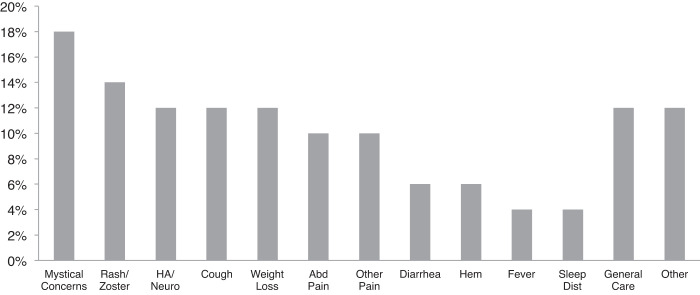
Participant-reported reasons for consulting a traditional healer. (A) Some participants provided more than one reason; therefore, the sum is greater than 100%. (B) Mystical concerns include mystical problems, mystical treatments, bad spirits, spiritual offerings, protective washing, and rituals. (C) Median time interval between the last consultation with a traditional healer and visit to the HIV clinic: 2 months (interquartile range [IQR], 1-5 months). HA, headache; Neuro, dizziness and numbness; Abd, abdominal; Other Pain, joint pain and diffuse pain; Hem, hemorrhoids; Sleep Dist, sleep disturbance including nightmares and insomnia.

The most commonly reported reason for consulting a TH was “mystical” concerns (18%), which included “mystical” problems, “mystical” treatments, “bad spirits,” spiritual offerings, protective washing, and rituals (Figure 1). Other specific reasons reported by ≥10% of participants included rash or zoster, headaches or other neurological symptoms such as dizziness or numbness, cough, weight loss, abdominal pain, and other pain, including joint pain and “diffuse pain.” Participants also sought care from THs for diarrhea, hemorrhoids, fever, nightmares, insomnia, and general care.

**Figure 2.**
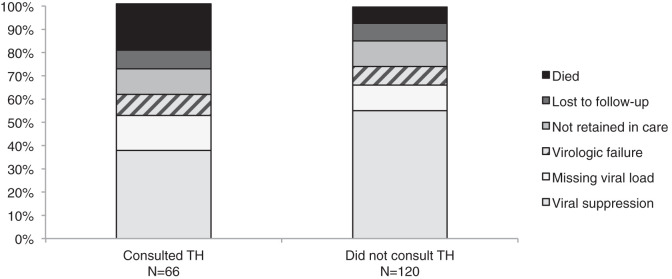
Outcomes according to consultation with a traditional healer (TH) prior to ART or during follow-up versus did not consult a TH.

A greater percentage of those who consulted a TH before the initiation of ART presented with a CD4 cell count < 200 cells/mm^3^ (44% versus 28%; *P* = 0.04) and WHO clinical stage 3 or 4 disease (64% versus 46%; *P* = 0.03) compared with those who did not consult a TH before the initiation of ART (Table 2). The median number of days between HIV diagnosis and ART initiation was not significantly different among those who had consulted a TH compared to those who had not consulted a TH (9 days [IQR 5–20] versus 11 days [IQR 6–29]; *P* = 0.35). Those who consulted a TH before ART initiation were less likely to disclose their HIV status to anyone (44% versus 65%; *P* = 0.04). Among those who consulted a TH before ART initiation, 22% died compared to 7% among those who did not consult a TH before ART initiation (*P* < 0.01) (Table 2A, Figure 2). Overall, 80% of deaths occurred within 6 months of enrollment. The median number of months from enrollment to death was 3.0 among those who consulted a TH compared to 4.5 among those who did not consult a TH (*P* = 0.21). Loss to follow-up, poor adherence to ART, and virologic failure did not differ among those who consulted a TH before ART initiation and those who did not. Poor adherence to ART (57% versus 4%; *P* < 0.01) and virologic failure (50% versus 12%; *P* = 0.03) were more common among those who consulted a TH 6 months after ART initiation compared to those who did not consult a TH 6 months after ART initiation (Table 2B).

**Table 2:** HIV outcomes according to consultation with a traditional healer (TH) prior to initiation of ART (A) and consultation with a TH after 6 months of ART (B)

A. Consultation with a TH prior to	All participants, *N* = 177	Consulted TH before treatment, *N* = 58	Did not consult TH before treatment, *N* = 119	
initiation of ART	*n* (%)	*n* (%)	*n* (%)	*P* value
CD4 count < 200 cells/mm^3 ^before treatment (*N* = 166)	55 (33.1)	23 (44.2)	32 (28.1)	0.04
Stage 3 or 4 disease at baseline (*N* = 157)	82 (52.2)	34 (64.2)	48 (46.2)	0.03
Disclosed HIV status (*N* = 109)	64 (58.7)	14 (43.8)	50 (64.9)	0.04
Lost to follow-up (*N* = 177)	14 (7.9)	5 (8.6)	9 (7.6)	0.78
Died (*N* = 177)	21 (11.9)	13 (22.4)	8 (6.7)	< 0.01
Poor adherence to ART (*N* = 105)	8 (7.6)	3 (10.0)	5 (6.7)	0.69
Virologic failure (*N* = 99)	13 (13.1)	4 (16.7)	9 (12.0)	0.51
B. Consultation with a TH after 6 months of ART	All participants, *N* = 107 *n* (%)	Consulted TH after 6 months, *N* = 7 *n* (%)	Did not consult TH after 6 months, *N* = 100 *n* (%)	*P* value
Poor adherence to ART (*N* = 103)	8 (7.8)	4 (57.1)	4 (4.2)	< 0.01
Virologic failure (*N* = 93)	13 (14.0)	3 (50.0)	10 (11.5)	0.03

Of the 186 participants, 91 achieved viral suppression after 12 months of ART, 15 had virologic failure, 22 died, and 58 did not have viral load outcome data. Of the 58 participants without viral load outcome data, 15 were lost to follow-up, 10 had migrated, 7 had transferred care, and 26 were known to be alive but were missing viral load data. Comparison of baseline characteristics among those with viral load outcome data to those without viral load outcome data indicated that those without viral load data were more likely to have been enrolled at the Ziguinchor site.

The strongest predictor of virologic failure was consulting a TH more than 6 months after ART initiation (odds ratio [OR], 7.43; 95% CI, 1.22–45.24) (Table 3). A sensitivity analysis in which the 15 participants who were lost to follow-up were categorized as virologic failures did not significantly change the findings described in Table 3, nor did a sensitivity analysis in which the 58 participants with missing viral load outcome data were categorized as virologic failures. The strongest predictors of mortality were consulting a TH before ART initiation (OR, 3.53; 95% CI, 1.25–9.94) and baseline CD4 count < 200 cells/mm^3^ (OR, 3.15; 95% CI, 1.12–8.89) (Table 4).

**Table 3:** Logistic regressions showing predictors of virologic failure after 12 months of antiretroviral therapy (ART) among HIV-infected individuals in Senegal

	Simple regressions			
	Odds ratio	95% CI		*P* value
Ziguinchor site (ref. Dakar)	2.40	0.79	7.33	0.12
Male (ref. female)	1.40	0.43	4.50	0.58
Age	1.04	0.98	1.10	0.19
Age category (ref. ≥ 45 years)				
> 45	3.70	1.12	12.23	0.03
Born in Senegal	0.46	0.13	1.66	0.24
Education (ref. no education)				
Any primary school	0.68	0.15	3.01	0.61
Any secondary school	1.14	0.27	4.83	0.86
Employed	0.94	0.19	4.68	0.94
Transportation time ≥ 90 minutes	0.48	0.14	1.64	0.24
Transportation cost to clinic (for every $0.10 increase)	1.27	0.60	2.66	0.53
Transportation cost ≥ $1.75	0.68	0.17	2.63	0.57
Consulted a traditional healer before ART initiation	1.47	0.41	5.27	0.56
Consulted a traditional healer after 6 months of ART	7.70	1.36	43.46	0.02
	Multiple regression, *N* = 91			
	OR	95% CI		*P* value
Age category (ref. ≤ 45 years)				
> 45	3.57	0.99	12.88	0.05
Consulted a traditional healer after 6 months of ART	7.43	1.22	45.24	0.03

**Table 4:** Logistic regressions showing predictors of mortality during the first 12 months of antiretroviral therapy (ART) among HIV-infected individuals in Senegal

	Simple regression			
	Odds ratio	95% CI		*P* value
Ziguinchor site (ref. Dakar)	1.20	0.47	3.05	0.71
Male (ref. female)	2.14	0.86	5.29	0.10
Age	1.04	1.00	1.08	0.09
Age category (ref. ≤ 45 years)				
> 45	1.15	0.42	3.15	0.78
Born in Senegal	0.78	0.27	2.28	0.64
Education (ref. no education)				
Any primary school	0.67	0.21	2.12	0.49
Any secondary school	1.13	0.36	3.49	0.84
Employed	1.69	0.51	5.58	0.39
Transportation time ≥ 90 minutes	1.27	0.49	3.30	0.62
Transportation cost to clinic (for every $0.10 increase)	1.17	0.59	2.34	0.66
Transportation cost ≥ $1.75	0.90	0.28	2.88	0.85
Consulted a traditional healer before ART initiation	4.01	1.56	10.33	< 0.01
Baseline CD4 count < 200 cells/mm^3^	3.68	1.34	10.07	0.01
	Multiple regression,* *N* = 161			
	Odds ratio	95% CI		*P* value
Male (ref. female)	1.63	0.57	4.67	0.36
Age category (ref. ≤ 45 years)				
> 45	0.85	0.27	2.75	0.79
Consulted a traditional healer before ART initiation	2.93	1.04	8.22	0.04
Baseline CD4 count < 200 cells/mm^3^	3.26	1.13	9.37	0.03
	Multiple regression,† *N* = 166			
	Odds ratio	95% CI		*P* value
Consulted a traditional healer before ART initiation	3.53	1.25	9.94	0.02
Baseline CD4 count < 200 cells/mm^3^	3.15	1.12	8.89	0.03

* Model includes variables associated with mortality in prior studies and variables identified as significant by simple regression.

† Model includes variables identified as significant by simple regression.

## DISCUSSION

In this prospective, longitudinal study conducted among HIV-positive individuals in Senegal, consultation with a TH was associated with poor outcomes along the entire HIV cascade of care. Consultation with a TH prior to initiation of ART was associated with presentation with advanced disease, non-disclosure of HIV status, and increased mortality, while continued consultation with a TH was associated with poor adherence to ART and virologic failure. Importantly, we found that consultation with a TH was not a risk factor for loss to follow-up. Our findings reveal multiple points along the HIV cascade of care where partnership between THs and biomedical providers could be of particularly high impact.

### TH-initiated HIV testing.

Although we did not assess the time interval between the first consultation with a TH and HIV diagnosis, individuals who consulted a TH before ART initiation were more likely to present with advanced disease. This finding suggests that consultation with THs may be contributing to delays in the diagnosis of HIV. This finding is consistent with our previous work in Senegal^10^^,^^12^ and studies conducted in Mozambique, Gabon, and Uganda, in which consultation with THs was associated with delays in HIV diagnosis and treatment.^13^^–^^15^ As such, recognizing that THs may be the first point of care for many individuals living with HIV in SSA, and engaging THs to conduct HIV testing may be a high-impact strategy to increase access to early HIV diagnosis, improve survival, and strengthen the HIV care cascade. Although partnerships with THs have been recognized by the WHO and the United Nations Programme on HIV and AIDS^2^^,^^5^ as a critical component to increasing access to HIV testing and treatment in SSA, there have been no published studies in which THs have been engaged to conduct HIV testing or to help facilitate HIV self-testing of their clients.

### TH education and referral.

Importantly, although the most common reason for consulting a TH was for “mystical concerns,” THs were also frequently consulted for symptoms consistent with advanced HIV, immunocompromised status, and opportunistic infections. This finding suggests that educating THs to recognize signs and symptoms that should prompt referral and facilitating referral to partner biomedical facilities may help to improve the clinical care and survival of PLHIV.

In a number of countries throughout SSA, training has been provided to THs to increase knowledge of HIV and HIV preventive practices^2^^,^^16^^,^^17^ and a few studies have explored partnering with THs to increase early referral and linkage to care.^18^^,^^19^ In a study conducted in South Africa, a 3.5-day HIV/AIDS training intervention was provided to THs, and knowledge, attitudes, and practices were compared before and after the intervention.^18^ Overall HIV knowledge, safe practices, and willingness to refer patients to biomedical facilities increased after the intervention. Interestingly, referral to and from other THs also increased after the intervention. Similarly, during a study conducted in Mozambique, an educational intervention was provided to THs and referral rates were evaluated before and after the intervention.^19^ THs received 3 days of training with the stated purposes of helping the THs to identify patients with HIV, tuberculosis, malaria, malnutrition, mental illness, and diarrhea and to provide information about the importance of early detection and referral for standard methods of treatment. After the training, the overall number of referrals increased by 35%. The findings from these promising studies warrant further investigation and evaluation in additional settings on a larger scale.

### THs as adherence support partners.

We found that the majority of individuals who consulted a TH before enrollment ceased to consult with THs after initiating ART. However, those who continued to consult THs were at increased risk of poor adherence to ART and virologic failure. To our knowledge, this is the first study to demonstrate that consultation with THs is associated with virologic failure after initiating ART.

Previous studies conducted in SSA have suggested that individuals who seek care from THs are at increased risk of poor ART utilization and adherence. During a study conducted in Tanzania, Uganda, and Zambia, consultation with a TH was associated with incomplete adherence, which was defined as having missed at least 48 consecutive hours of ART during the past 3 months.^20^ In Cameroon, consultation with a TH was associated with increased risk of self-reported treatment interruption lasting more than 2 consecutive days.^21^ Among HIV-positive individuals in South Africa, those who sought care from both THs and biomedical facilities were less likely to use ART compared with those who sought care from biomedical facilities alone.^22^

Given that some participants continue to seek care from THs after initiating ART, engaging THs as adherence support partners may be an effective strategy to improve patient adherence to ART and decrease virologic failure. One study conducted in Mozambique explored the feasibility of engaging THs as adherence support partners for individuals with newly diagnosed HIV.^23^ The FHI360 Adherence Support Workers program was adapted for use with THs using theater presentations and focus groups, and the acceptability and feasibility of the program were evaluated. Proposed adherence support activities included, reassuring patients of the allopathic diagnosis and treatment, providing medication pickup for patients too weak to travel, providing home-based counseling about HIV adherence, nutrition, and relationships, and providing clinic-based advocacy for PLHIV. Advocacy included clarification of any instructions and explanations provided by clinicians and advocating in defense of patients’ rights if poor treatment was witnessed. The program was found to be acceptable and feasible by clinicians, healers, community members, and the majority of PLHIV. However, the efficacy of the modified adherence support program and its impact on patient outcomes were not evaluated. Randomized clinical trials that evaluate adherence to ART and virologic failure should be performed to determine the impact of THs as adherence support partners on HIV outcomes.

### Engaging THs to seek out those lost to follow-up.

Commonly reported reasons for which individuals seek care from THs rather than biomedical care are convenience, distance to a biomedical facility, costs, health beliefs, spiritual beliefs, trust in THs, mistrust of biomedical treatments, and influence of family members and friends.^24^^–^^33^ As such, compared with biomedical providers, THs may have greater physical access to those lost to follow-up because they live and practice in the villages where they reside, or they may have greater social access because of established belief systems, trust, and the influence of peers. In these settings, THs may be well-positioned to reach those who are lost to follow-up and guide them to re-engage in care. To our knowledge, there have been no published studies in which THs were engaged to seek out those lost to follow-up and improve retention in care.

### Educating THs to address HIV-related stigma.

Nondisclosure of HIV status is considered a “proximate consequence” of self-stigmatization^34^^,^^35^ which contributes to depression, decreased social engagement, and decreased quality of life.^34^^–^^40^ Furthermore, nondisclosure of HIV status is associated with poor HIV outcomes, including decreased adherence to ART and increased risk of HIV transmission.^34^^,^^39^^,^^40^ In this study, non-disclosure of HIV status was more common among individuals who consulted a TH, suggesting that individuals who seek care from THs may be suffering from a greater degree of self-stigmatization. Previous studies have found that anticipated stigma associated with the utilization of HIV-specific clinics is a barrier to HIV testing and linkage to care, and drives individuals to seek care from THs rather than biomedical facilities.^41^^,^^42^ Stigma has also been associated with the belief that HIV is caused by spiritual and supernatural forces and is inverse to AIDS-related knowledge.^43^ Training THs to serve as peer educators may be an important component of a strategy to address HIV-related stigma and discrimination at both the community and individual levels.

The strengths of our study include its longitudinal study design, the use of multiple sources of data, and the enrollment of individuals in two different regions of the country to capture a sample that is more representative of the HIV-positive population in Senegal. Our study was limited by its sample size, incomplete data regarding virologic outcomes, and limited qualitative data regarding health beliefs, health-seeking behaviors, and perspectives of THs.

Seeking care from THs is common among PLHIV in Senegal, and is associated with poor outcomes along the entire HIV cascade of care. Our findings reveal multiple opportunities to strengthen the HIV care cascade through partnership between THs and biomedical providers, including TH-initiated HIV testing, TH education and referral, engaging THs as adherence support partners, engaging THs to seek out those lost to follow-up, and educating THs to address HIV-related stigma. Future studies to evaluate the impact of these strategies on HIV outcomes are warranted.
